# Exploring the relationships among interpersonal competence, perceived stress and cognitive reappraisal in Chinese PAP recruits: a longitudinal cross-lag study

**DOI:** 10.3389/fpsyg.2025.1672339

**Published:** 2026-01-09

**Authors:** Liyun Sun, Min Li

**Affiliations:** 1 Department of Military Psychology, Faculty of Medical Psychology, Army Medical University (Third Military Medical University), Chongqing, China; 2 Department of Medical Psychology, Liaoning Province Hospital of the Chinese People’s Armed Police Force, Shenyang, Liaoning, China

**Keywords:** cognitive reappraisal, interpersonal competence, longitudinal cross-lag study, perceived stress, PAP

## Abstract

**Background:**

The associations among interpersonal competence (IC), cognitive reappraisal (CR) and perceived stress (PS) among recruits of the Chinese People’s Armed Police Force (PAP) are poorly understood. The aim of this study is to clarify the relationships among these variables over time.

**Methods:**

A three-wave longitudinal study involving 300 recruits of the PAP surveyed over 3 months was conducted. The Chinese version of the Perceived Stress Scale (CPSS), the Brief Interpersonal Competence Questionnaire (ICQ-15), and the cognitive reappraisal scale, excerpted from the Emotion Regulation Questionnaire (ERQ), were chosen to assess PS, IC and CR. Longitudinal connections between these variables were examined via structural equation analysis.

**Results:**

Our results revealed that time 1 IC negatively predicted time 2 PS and that time 1 PS negatively predicted time 2 IC. Time 1 IC positively predicted time 2 CR, time 2 IC positively predicted time 3 CR, time 1 PS negatively predicted time 2 CR, and time 2 PS negatively predicted time 3 CR.

**Conclusion:**

These findings highlight the role of IC in initiating stress and cognitive reappraisal. In this study, IC decreased PS and improved CR temporally. Therefore, the interpersonal competence of PAP recruits needs to be cultivated.

## 1 Introduction

Currently, most soldiers are not engaged in wars or conflicts (Cai et al., 2017), but they face many other types of stressors. Thus, stress among soldiers is a growing health concern. Local youth can take on the role of recruits on their way to becoming soldiers; during this transition, they face various pressures from their environment, including military training, planning for the future and interpersonal relationships. Civilians can successfully cope with this pressure and become qualified soldiers. However, some recruits experience maladjustment and mental or emotional disorders such as depression, anxiety, posttraumatic stress disorder (Kutz and Dekel, 2006), and even suicidal behavior (Anglemyer et al., 2016). Thus, preventing recruits from developing mental disorders and reducing high-stress states are particularly important.

### 1.1 Relationship between interpersonal competence and perceived stress

A comprehensive understanding of stress dynamics can be grounded in Lazarus and Folkman’s (1984) transactional model of stress and coping (Folkman et al., 1986). This model proposes that stress is determined not only by external events but also by the dynamic interplay between individuals and their environment, mediated by cognitive appraisals. The model differentiates between primary appraisal (evaluating whether an event is threatening or challenging) and secondary appraisal (assessing one’s resources to cope with the demand). Within this framework, successful adaptation depends on the availability and effectiveness of coping resources and strategies. Interpersonal competence (IC), defined as the ability to send and receive verbal and nonverbal messages appropriately and effectively (Kanning, 2006), is a crucial personal resource that can influence this transactional process. IC facilitates the acquisition of social support—a well-established buffer against stress (Alsubaie et al., 2019). By enabling individuals to express needs and expand their social networks (Buhrmester et al., 1988), IC can positively influence both primary appraisal (by reducing the perceived threat of social situations) and secondary appraisal (by increasing perceived access to support). While studies have linked IC to lower depression and loneliness in other populations (Bird et al., 2018), its longitudinal relationship with PS among military recruits, particularly within a culture that emphasizes group harmony, remains underexplored.

### 1.2 Relationship between emotion regulation and perceived stress

Emotion regulation strategies are understood as cognitive approaches to managing the intake of emotionally arousing information and encompass a broad range of cognitive, behavioral, emotional, and physiological responses (Jinyao et al., 2012). Cognitive emotion regulation strategies are divided into adaptive strategies and maladaptive strategies. Reappraisal (Appleton et al., 2013), which is regarded as an adaptive strategy, involves altering how one thinks about an emotion by eliciting the situation to change its emotional impact. Suppression, which is considered a maladaptive strategy, involves inhibiting emotional expression in response to an emotion-eliciting event. a growing body of research suggests that cognitive appraisals (CRs) are powerful tools that help shift negative stress states to more positive states (Jamieson et al., 2013). Emotion regulation, is not only a critical factor in the stress process but can also be influenced by stress. Kinner et al. (2014) demonstrated that stress and the stress hormone cortisol facilitate emotion regulatory processes, especially via cognitive reappraisal. Langer et al. (2020) found that acute stress led to an improvement in reappraisal among men, and they reported that stronger pupil dilation further demonstrated that stressed males were cognitively more engaged during reappraisal, which in turn might have led to better emotion regulatory outcomes. Initial studies provided mixed evidence showing either beneficial or impairing effects of stress on the regulation of cognitive emotion depending on the timing of stress, sex or regulatory strategy. In the Chinese armed forces with all the male soldiers, whether CR affects PS or whether it affects CR is the second objective of our study.

### 1.3 Relationship between interpersonal competence and emotion regulation

Interpersonal competence (IC) and emotion regulation are fundamentally interconnected. Gross’s (1998) process model delineates the emotion generation process and posits that regulation can occur at different points along this timeline. Interpersonal competence can be understood as a key resource that facilitates effective regulation across these various stages. In particular, during the cognitive change stage, IC provides essential social-cognitive tools. The ability to understand diverse perspectives and engage in effective verbal exchange furnishes individuals with alternative interpretations of emotionally charged events, thereby facilitating a more adaptive reappraisal process. There is some empirical evidence to support the relationship between interpersonal competence and emotion regulation. For example, Mansi Varma reported that cognitive reappraisal was moderately positively correlated with the ability to send clear messages and life satisfaction among young participants (Mansi Varma, 2024), and Aqib and Rizwi (2020) reported that emotion regulation and interpersonal communication were positively correlated with each other. However, existing studies are limited to exploring the correlation between these two factors, and their underlying relationships are not clear. This is especially true for Chinese PAP recruits.

### 1.4 The current study

Most research has focused on cross-sectional studies, and the relationships among the effects of IC, PS and CS on Chinese PAP recruits are unclear. Thus, a longitudinal study was designed to explore the associations among the effects of IC, PS and CS on Chinese PAP recruits. We hypothesize that (1) IC negatively predicts perceived stress among the recruitment of Chinese PAP recruits. (2) IC positively predicts cognitive reappraisal among Chinese PAP recruits. (3) Cognitive reappraisal negatively predicts perceived stress among the recruitment of Chinese PAP recruits.

## 2 Methods

### 2.1 Participants

This study was reviewed and approved by the Medical Ethics Committee of the Liaoning Province Hospital of the Chinese PAP (20250716001). A total of 300 recruits who enlisted in a certain policeman force in March 2024 participated in our survey. Participants completed measures at three time points over a 3-month period. This time interval (Junrun et al., 2014) was selected so that we can examine the stress variation changes. Due to some soldiers ‘official duty or hospitalization, finally, 275 valid questionnaires were obtained. All the participants were male and aged between 18 and 22 years (*M* = 19.03 years, SD = 2.16). Thirty-two people had a high school education, and 243 people had an associate degree or above. Approximately 88.14% of the participants came from intact families, and 11.86% came from a single parent. To examine the mechanism of missing data, Little’s Missing Completely at Random (MCAR) test was performed (Little, 1988). the result was non-significant[χ^2^(25) = 27.80, *p* = 0.31], suggesting that the data were likely missing completely at random, thereby justifying the use of Full Information Maximum Likelihood (FIML) estimation for handling missing values.

### 2.2 Measures

#### 2.2.1 Chinese version of the perceived stress scale

Perceived stress was evaluated by using the Chinese version of the 14-item Perceived Stress Scale (Xu et al., 2023). Items (e.g., upset by the occurrence of unexpected events) were rated from 0 (never) to 4 (very often), and seven items (4, 5, 6, 7, 9, 10, 13) were reverse scored (e.g., I can often master time management). The total score was the sum of all the items with reverse coding of the relevant items and ranged from 0 to 56. The higher the total score is, the greater the perceived stress. In the current study, the Cronbach’s α coefficients were 0.92, 0.82, 0.87 separately.

#### 2.2.2 Brief interpersonal competence questionnaire

The Chinese version of the Brief Interpersonal Competence Questionnaire was used to assess five aspects (Huang et al., 2022): initiative [e.g., introducing yourself to someone you might like to know (or date)], negative assertion (e.g., telling a companion that he or she has done something to hurt your feelings), disclosure (e.g., letting a new companion get to know the “real” you), emotional support (e.g., when a close companion needs help and support, being able to give advice in ways that are well received), and conflict management (e.g., being able to admit that you might be wrong when a disagreement with a close companion begins to build into a serious fight). Responses were given on a 5-point scale (1 = very difficult, 5 = very easy). Higher scores indicate stronger interpersonal competence. The Cronbach’s coefficients in this study were 0.92, 0.95, 0.93 separately.

#### 2.2.3 Emotion regulation questionnaire

The cognitive reappraisal scale (when I want to feel more *positive* emotions (such as joy or amusement), I *change what I am thinking about*) is derived from the emotion regulation strategy questionnaire developed by Gross (Preece et al., 2020). It consists of six items and uses a seven-point rating from 1 (almost never) to 7 (almost always). A higher score denotes greater use of a specific emotion regulation strategy. In this study, a cognitive reappraisal scale was used, and the Cronbach coefficients were 0.84, 0.89, and 0.87.

### 2.3 Research procedure

During each assessment, the same batch of questionnaires was group administered in meeting rooms of 50 recruits under the supervision of trained psychologists to ensure the accuracy of the survey. Before completing the questionnaire, all participants were instructed to respond based on their actual circumstances. They were assured that their responses would be collected anonymously, used solely for scientific research purposes, and kept strictly confidential.

### 2.4 Data processing

The distribution of scales was uniformly carried out by two trained psychologists. The collected questionnaires were input by professionals. The researcher encoded the 275 × 3 valid questionnaires, and descriptive analysis and correlation analysis were conducted with SPSS 28.0. Furthermore, cross-lagged models were constructed with Mplus.

## 3 Results

### 3.1 Descriptive common method bias test

A common method bias (CMB) test was conducted on all variable items collected at T1 using Harman’s single-factor test (Podsakoff et al., 2003). The results revealed that there were 9 factors with eigenvalues greater than 1, and the first factor accounted for 28.15% of the variance, which is below the critical threshold of 40%. This indicates that no significant common method bias was present.

### 3.2 Descriptive statistics and correlation analysis of variables

Table 1 presents the means, standard deviations, and zero-order bivariate correlations among the variables at Times 1, 2 and 3.

**Table 1:** Descriptive statistics and correlations among the variables.

Variable	*M* ± SD	1	2	3	4	5	6	7	8	9
1 PS1	14.96 ± 5.52	1								
2 PS2	14.14 ± 6.11	0.58^**^	1							
3 PS3	13.42 ± 5.82	0.38^**^	0.39^**^	1						
4 Ic1	53.65 ± 10.02	−0.44^**^	−0.47^**^	−0.22^**^	1					
5 Ic2	56.89 ± 11.98	−0.41^**^	−0.59^**^	−0.29^**^	0.68^**^	1				
6 Ic3	59.54 ± 11.50	−0.27^**^	−0.31^**^	−0.40^**^	0.40^**^	0.43^**^	1			
7 CR1	30.47 ± 5.17	−0.30^**^	−0.15^**^	−0.16^**^	0.41^**^	0.25^**^	0.17^**^	1		
8 CR2	32.09 ± 6.03	−0.28^**^	−0.35^**^	−0.17^**^	0.38^**^	0.48^**^	0.17^**^	0.42^**^	1	
9 CR3	33.77 ± 6.35	−0.28^**^	−0.26^**^	−0.45^**^	0.21^**^	0.27^**^	0.41^**^	0.29^**^	0.38^**^	1

**p < 0.01.

Repeated-measures ANOVA (Mauchly, *p < 0.05*; multivariate test, *F* = 29.04; *p* < 0.05) revealed a significant decreasing trend in stress scores over the three-month period (*F* = 8.056; *p* < 0.05). Specifically, there was no significant difference in stress between T2 and T3, whereas significant differences were observed between all the other time points (*p* < 0.05). Conversely, the IC scores and CR scores significantly increased at each time point (*F* = 34.785, *p* < 0.05). Furthermore, positive correlations from T1 to T2 and from T2 to T3 (*p* < 0.05) were observed for PS, IC and CR (*p* < 0.05). Thus, there were contemporaneous correlations among IC, PS, and CR. the intercorrelations among the time 1 variables were also significant. IC was negatively correlated with concurrent PS (*r* = −0.44, *p* < 0.01). IC was positively correlated with the concurrent CR (*r* = −0.41, *p* < 0.01). RC was negatively correlated with the concurrent PS. When time 1 effects were controlled for, the time 2 variables were also moderately correlated with each other. When time 1 and time 2 effects were controlled for, the time 3 variables were also moderately correlated with each other. The results indicated a synchronous relationship between IC, PS and CR, as shown in Table 1. T1 IC and T2 PS were significantly negatively correlated (*r* = −0.47, *p* < 0.05), as were T1 PS and T2 IC (*r* = −0.41, *p* < 0.05). Moreover, the correlation coefficient between T1 IC and T2 PS was slightly greater than the correlation coefficient between T1 PS and T2 IC. T1 IC and T2 CR were significantly positively correlated (*r* = 0.38, *p* < 0.05), as were T1CR and T2 IC (*r* = 0.27, *p* < 0.05). Moreover, the correlation coefficient between T1 IC and T2 CR was slightly greater than the coefficient between T1 CR and T2 IC. T1 CR and T2 PS were slightly negatively correlated (*r* = −0.15, *p* < 0.05), as were T1 PS and T2 CR (*r* = −0.28, *p* < 0.05). T2 CR and T3 PS were slightly negatively correlated (*r* = −0.15, *p* < 0.05), as were T2 PS with T3 CR (*r* = −0.26, *p* < 0.05).

### 3.3 Cross-lagged panel model of interpersonal competence and perceived stress

On the basis of the correlations, we estimated the full cross-lag model. We also conduct the tests of configural, metric, and scalar invariance (Wang et al., 2021; Cheung and Rensvold, 2002) as shown in Table 2. Metric invariance was supported when compared to the configural model (ΔCFI = +0.004, ΔRMSEA = +0.001). However, scalar invariance was not supported, as the fit deteriorated significantly when constraints were added (ΔCFI = 0.013) (Little, 2024). The full model fit the data well; χ^2^ = 6.64, GFI = 0. 99, TLI = 0.96, RMSEA = 0.07, 90% CI [0.00, 0.14], SRMR = 0.02.

**Table 2:** Tests of configural, metric and scalar invariance.

Model	χ^2^	df	CFI	RMSEA95%[CI]	ΔCFI	ΔRMSEA
Perceived stress						
Configural	2436.67	817	0.817	0.086 [0.082, 0.090]		
Metric	3085.00	843	0.813	0.097 [0.096, 0.104]	0.004	0.001
Scalar	3229.31	868	0.800	0.101 [0.097, 0.105]	0.013	0.004
Interpersonal competence						
Configural	1686.15	880	0.910	0.089 [0.088, 0.111]		
Metric	2469.50	971	0.906	0.078 [0.074, 0.081]	0.04	0.011
Scalar	2513.80	943	0.905	0.078 [0.074, 0.082]	0.001	0.000
Cognitive reappraisal						
Configural	248.19	133	0.956	0.056 [0.045, 0.067]		
Metric	281.34	143	0.951	0.060 [0.049, 0.070]	0.005	−0.004
Scalar	279.79	152	0.940	0.055 [0.045, 0.066]	0.009	0.005

χ^2^, chi-square statistic; CFI, Comparative Fit Index; RMSEA, Root Mean Square Error of Approximation.

**Figure 1:**
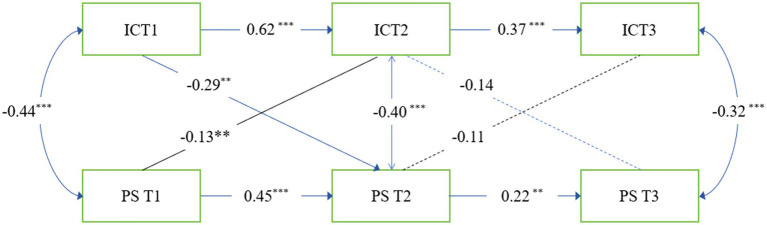
Cross-lagged panel model of IC and PS. PS, perceived stress; IC, interpersonal competence; **p < 0.01, ***p < 0.001.

As shown in Figure 1, interpersonal competence at T1 negatively predicted PS at T2 (β = −0.29, *p* < 0. 01), and PS at time 1 also negatively predicted IC at Time 2 (β = −0.13, *p* < 0.01). However, the coefficient of the former is larger than that of the latter. Furthermore, IC at T2 did no negatively predict T3 PS, and T2 PS did not negatively predict T3 IC. Path coefficients for the relationships between perceived stress and *interpersonal competence* are shown in Table 3.

**Table 3:** Path coefficients for the relationships among interpersonal competence, perceived stress and cognitive reappraisal.

Variables	PS1 ≥ IC2	PS2 ≥ IC3	IC1 ≥ PS2	IC2 ≥ PS3	IC1 ≥ CR2	IC2 ≥ CR3	CR1 ≥ IC2	CR2 ≥ IC3	CR1 ≥ PS2	CR2 ≥ PS3	PS1 ≥ CR2	PS2 ≥ CR3
β	−0.13*	−0.11	−0.29***	−0.14	0.24***	0.15*	0.03	0.05	−0.01	−0.05	−0.16*	−0.16*
SE	0.05	0.07	0.05	0.07	0.06	0.07	0.05	0.06	0.05	0.06	0.06	0.06
95% CI	[−0.23, −0.03]	[−0.25, 0.03]	[−0.38, −0.18]	[−0.28, 0.01]	[0.20, 0.44]	[0.01, 0.29]	[−0.07, 0.13]	[−0.07, 0.17]	[−0.11, 0.09]	[−0.17, 0.07]	[−0.28, −0.04]	[−0.28, −0.04]

*p < 0.05; ***p < 0.001.

### 3.4 Cross-lagged panel model of interpersonal competence and cognitive reappraisal

**Figure 2:**
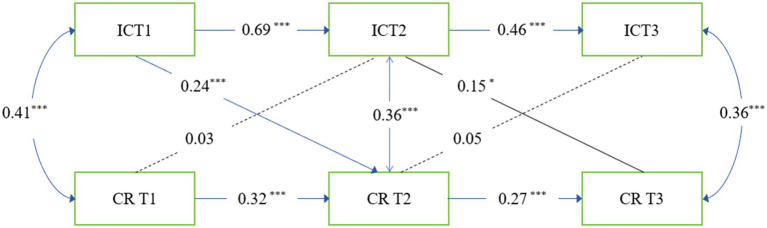
Cross-lagged panel model of IC and CR. IC, interpersonal competence; CR, cognitive reappraisal; *p < 0.05, **p < 0.01, ***p < 0.001.

The longitudinal measurement model showed an excellent fit to the data. We also conduct the tests of configural, metric, and scalar invariance as shown in Table 2. For Interpersonal Competence, both metric (ΔCFI = −0.004, ΔRMSEA = −0.011) and scalar invariance (ΔCFI = −0.001, ΔRMSEA = 0.000) were supported, with all changes well within acceptable thresholds.as shown in Table 2. The full model fit the data well; χ^2^ = 8.13, df = 3, GFI = 0. 98, TLI = 0.93, RMSEA = 0.08, 90% CI [0.01, 0.15], SRMR = 0.02.as illustrated in Figure 2, the IC at time 1 positively predicted the CR at time 2, and the IC at time 2 positively predicted the CR at time 3. However, time 1 CR could not positively predict time 2 IC and time 2 CR could not positively predict time 3 IC. Path coefficients for the relationships between perceived stress and *cognitive reappraisal* are shown in Table 3.

### 3.5 Cross-lagged panel model of cognitive reappraisal and perceived stress

**Figure 3:**
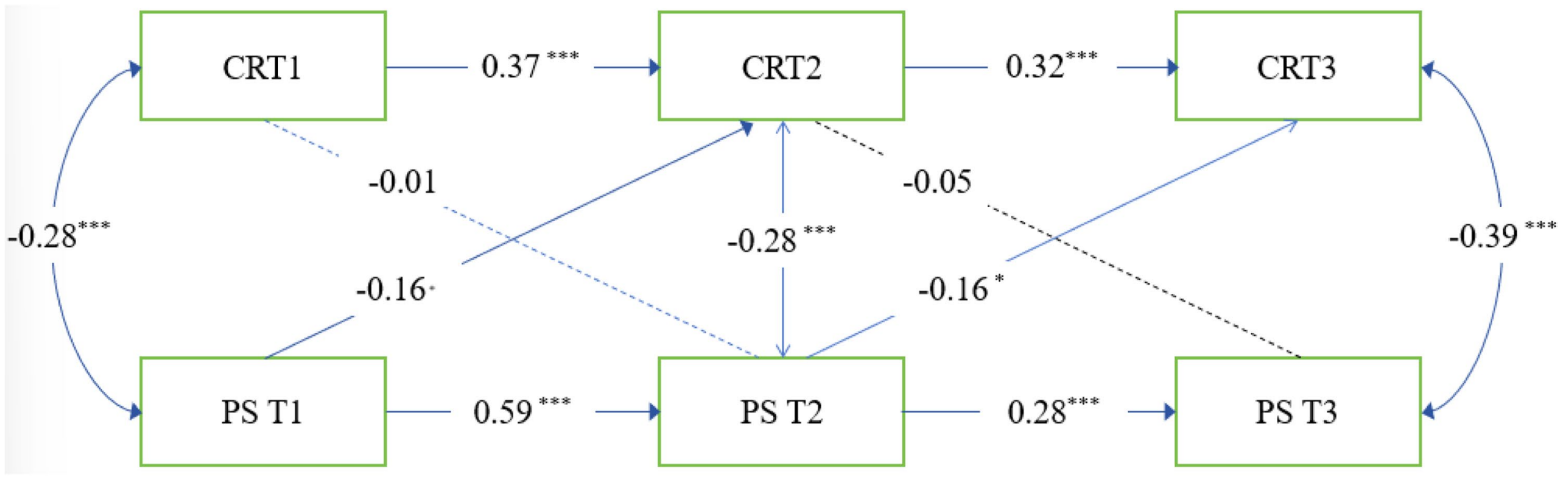
Cross-lagged panel model of PS and CR. IC, interpersonal competence; PS, perceived stress; *p < 0.05, **p < 0.01, ***p < 0.001.

On the basis of the correlations, we estimated the full cross-lag model. We also conduct the tests of configural, metric, and scalar invariance as shown in Table 2. For Cognitive Reappraisal, metric (ΔCFI = −0.005, ΔRMSEA = −0.004) and scalar invariance (ΔCFI = −0.009, ΔRMSEA = 0.005) were also supported. The full model fit the data well; χ2 = 9.72, *df* = 3, GFI = 0. 98, TLI = 0.90, RMSEA = 0.08, 90% CI [0.03, 0.16], SRMR = 0.03. As illustrated in Figure 3, time 1 PS negatively predicted time 2 CR, and time 2 PS negatively predicted time 3 CR. However, time 1 CR did not negatively predict time 2 PS, and time 2 CR did not negatively predict time 3 PS. path coefficients for the relationships between perceived stress and cognitive reappraisal are shown in Table 3.

## 4 Discussion

### 4.1 Trends in interpersonal competence, perceived stress and cognitive reappraisal

The present study investigated the relationships among the IC, PS and CR among Chinese PAP recruits. Firstly, we found that the stress level of the recruits gradually decreased, and the highest stress period occurred 1 month after they joined the army. Solders are most likely to be under high stress levels when they are exposed to a traumatic event. Secondly, IC and CR tended to increase over 3 months. The recruits’ IC and CR scores were the highest after 3 months of training. We consider that the initial surge in perceived stress among recruits during the first month can be attributed primarily to the profound psychosocial transition to a novel and demanding military environment. This period is characterized by the simultaneous challenges of environmental unfamiliarity, stringent disciplinary standards, and the complexity of new social hierarchies, collectively constituting a significant adaptive burden. As time progressed, a marked reduction in stress levels was observed, a phenomenon largely driven by the process of psychological acclimatization. Crucially, this adaptation was facilitated by the development of interpersonal competencies and the consequent consolidation of social support networks within the unit. The improvements in interpersonal and cognitive reappraisal skills are attributed to both structured military training, which provides deliberate practice, and the recruits’ innate propensity for psychological adaptation in demanding environments.

### 4.2 The impact of interpersonal competence on perceived stress

Cohen et al. (1986) provided evidence of a stress-buffering role of the perceived availability of social support. Social skill factors can predict the development of both social support and friendship formation (Buhrmester et al., 1988). Our longitudinal data revealed that time 1 IC can significantly predict time 2 PS. IC includes initiative, negative assertion, disclosure, emotional support and conflict management. People with stronger proactive communication may have a stronger social network (Cleary et al., 2023); thus, when facing pressure, through positive interaction, they can change their cognition of pressure and reduce their response to it. People who are comfortable with disclosing their feelings or offer help may obtain more support and thus experience less stress (Zhang, 2017; Zhen et al., 2021). Effective conflict management emphasizes the ability to address disagreements constructively and find mutually beneficial solutions (Ahmad and Chowdhury, 2022), which is essential for social integration, building a supportive network and reducing stress. Conversely, individuals with low levels of IC are less likely to have an opportunity to communicate their negative feelings with others or seek help, which could intensify stress symptoms (Forbes and Roger, 1999; Işık and Kaya, 2022; Spitzberg and Canary, 1985). These findings are consistent with those of previous studies (Wilks and Spivey, 2010). Our longitudinal data further revealed that higher perceived stress at T1 predicted lower interpersonal competence at T2. we considered that the initial, overwhelming stress of military transition likely temporal consumed substantial amounts of cognitive and emotional resources, resulting in recruits having a limited capacity for attending to social cues, exercising empathy, or engaging in proactive communication. Consequently, this resource scarcity impeded the normal development of interpersonal skills during the critical early phase of training.

We also found in our model that the path from interpersonal competence to subsequent stress is considerably stronger than the reverse path from stress to subsequent interpersonal competence. This finding indicates that interpersonal competence is an important protective factor against stress. These findings suggest that in the face of stressful situations, we can reduce the level of stress by improving interpersonal competence among PAP soldiers.

### 4.3 Impact of interpersonal competence on cognitive reappraisal

The results of the present study revealed that time 1 IC can significantly predict time 2 CR and that time 2 IC can significantly predict time 3 CR. firstly, IC contains five domains, and the initiation of interactions and assertiveness have commonly been studied in behavioral research on assertiveness (Lipton and Nelson, 1980; Borthakur and Sudhesh, n.d.; Joshy, 2023). Some studies (Sari and Priyambodo, 2022) have revealed a significant positive relationship between emotion regulation and assertiveness among students. Therefore, soldiers who are interpersonally competent are more likely to have high CR. secondly, soldiers with higher scores in IC are more likely to be willing to talk to others and effectively communicate their feelings and difficulties (Batenburg and Das, 2014). When individuals share their emotions, they have an opportunity to recount what happened and relieve their emotions and feelings, which provides space to help dissipate their negative emotions and improve their cognitive reappraisal (Batenburg and Das, 2014). Finally, when individuals share their stress proactively, they know that others have the same experience and that they understand that they are not facing difficulties alone. Furthermore, they can exchange methods and insights to address difficulties with each other. Afterward, they gain a greater sense of control over the situation and change their cognition. These results suggest that individuals’ interpersonal competence can improve the level of cognitive reappraisal among recruits (Efrat and Zait, 2022).

### 4.4 The impact of cognitive reappraisal on perceived stress

In this study, we found that perceived stress at time 1 could negatively predict cognitive reappraisal at time 2. However, the coefficients were slight, and we did not find that cognitive reappraisal could negatively predict perceived stress. We consider that it is also possible that the relationships among these factors involve mediating or moderating effects.

### 4.5 Limitations

Several limitations of this study should be noted. First, all of the participants in this study were new Chinese soldiers in Liaoning Province, and all the participants were male. It is unclear to what extent our findings can be generalized to recruits in other countries. Second, the effect sizes of the tests for the cross-lag relations of IC, PS, and CR were not large in the present study. Third, given the strong associations between IC, PS, and CR, more complicated pathways are likely. Future research should test more complicated pathways and explore potential mediating pathways among these variables. Fourth, we did not consider social desirability bias. At last, we use the CLPM, which conflates within-person between-person effects. This may lead to biased estimates. The Random Intercept Cross-Lagged Panel Model (RI-CLPM) will be considered for examining dynamic relationships among variables in future research.

## 5 Conclusion

Despite these limitations, our findings indicate that recruits who experienced higher levels of IC are more likely to experience higher levels of CR and lower levels of stress in the future. These findings suggest that helping recruits improve their IC may be useful for preventing stress and improving CR. Improving IC may be one way to prevent stress and improve CR in the long term.

## Data Availability

The original contributions presented in the study are included in the article/supplementary material, further inquiries can be directed to the corresponding author.
